# High-frequency rTMS modulates emotional behaviors and structural plasticity in layers II/III and V of the mPFC

**DOI:** 10.3389/fncel.2022.1082211

**Published:** 2022-12-13

**Authors:** Marco Cambiaghi, Carmenrita Infortuna, Francesca Gualano, Amir Elsamadisi, Wasib Malik, Mario Buffelli, Zhiyong Han, Ramon Solhkhah, Florian P. Thomas, Fortunato Battaglia

**Affiliations:** ^1^Department of Neurosciences, Biomedicine and Movement Sciences, University of Verona, Verona, Italy; ^2^Department of Biomedical and Dental Sciences, Morphological and Functional Images, University of Messina, Messina, Italy; ^3^Department of Medical Sciences, Hackensack Meridian School of Medicine, Nutley, NJ, United States; ^4^Department of Psychiatry, Hackensack Meridian School of Medicine, Nutley, NJ, United States; ^5^Department of Neurology, Hackensack Meridian School of Medicine, Nutley, NJ, United States; ^6^Department of Neurology, Hackensack University Medical Center, Hackensack, NJ, United States

**Keywords:** repetitive transcranial magnetic stimulation, spine density, dendritic complexity, medial prefrontal cortex, emotional behavior

## Abstract

Repetitive transcranial magnetic stimulation (rTMS) is a noninvasive neuromodulation technique, and it has been increasingly used as a nonpharmacological intervention for the treatment of various neurological and neuropsychiatric diseases, including depression. In humans, rTMS over the prefrontal cortex is used to induce modulation of the neural circuitry that regulates emotions, cognition, and depressive symptoms. However, the underlying mechanisms are still unknown. In this study, we investigated the effects of a short (5-day) treatment with high-frequency (HF) rTMS (15 Hz) on emotional behavior and prefrontal cortex morphological plasticity in mice. Mice that had undergone HF-rTMS showed an anti-depressant-like activity as evidenced by decreased immobility time in both the Tail Suspension Test and the Forced Swim Test along with increased spine density in both layer II/III and layer V apical and basal dendrites. Furthermore, dendritic complexity assessed by Sholl analysis revealed increased arborization in the apical portions of both layers, but no modifications in the basal dendrites branching. Overall, these results indicate that the antidepressant-like activity of HF-rTMS is paralleled by structural remodeling in the medial prefrontal cortex.

## Introduction

Major depressive disorder (MDD) is a prevalent medical condition causing substantial disability, depressed mood, limited sleep, decreased interest, impaired cognitive functioning, and altered appetite. Numerous pharmacological therapies have been used to treat MDD, but they are effective in only 50%–60% of MDD patients, and moreover, there is a very high relapse rate during the treatment (Dodd et al., 2018). To add to that, those patients who gave good responses to these medications reported unpleasant side effects including headache, nausea, gastrointestinal upset, and sexual dysfunctions (Dodd et al., 2018). For these reasons, there has been growing interest in the development of non-pharmacological methods to treat MDD. Non-invasive brain stimulation techniques, one of the fastest-growing fields in medicine, have received special attention as potential clinical tools (George et al., 2022). Among these techniques is repetitive transcranial magnetic stimulation (rTMS), which noninvasively excites neurons by rapidly changing magnetic fields (Barker et al., 1985). In humans, high-frequency (HF)-rTMS (5–25 Hz) applied over the prefrontal cortex has been associated with antidepressant effects and is now used as a treatment option for drug resistant depression (Somani and Kar, 2019). Based upon the frontal electroencephalographic asymmetry in depression and the hypothesis that the patient’s emotional reactivity is associated with the left frontal lobe hypofunction (Davidson, 1992; Allen et al., 2004), HF-rTMS of the left dorsolateral prefrontal cortex (DLPFC) is now the approved protocol to treat drug-resistant depression (George et al., 2010). Although most of the clinical n trials have Despite clinical evidence, the underlying mechanisms of how HF-rTMS induces the anti-depression effect are poorly known and the investigation of new brain targets for neuromodulation may optimize the therapeutic use of the technique.

The medial prefrontal cortex (mPFC) is an important area that modulates the behavioral response to stress, decision-making, memory, emotional, and inhibitory control (Riga et al., 2014). Previous studies in both humans with MDD and animal models demonstrated abnormal activation of mPFC circuits is linked to the development of depressive-like behaviors (Vialou et al., 2014; Marcus et al., 2020), rumination, and anhedonia (Williams, 2016). In addition, optogenetic stimulation of mPFC pyramidal neurons results in antidepressant effects (Covington et al., 2010). The functional contribution of mPFC networks to the development of MDD is further supported by studies indicating that chronic stress leads to synapse loss, reduced spine density, and decrease the apical dendritic length of layer II/III pyramidal neurons (Liu and Aghajanian, 2008; Csabai et al., 2018). These structural changes in the prefrontal cortex contribute to the pathophysiology of depressive disorders (Duman et al., 2016). Importantly, it appears that the administration of antidepressants blocks the effects of chronic stress and restores normal cortical synaptogenesis (Duman et al., 2016; Holmes et al., 2022). The prelimbic cortex, which is an mPFC subregion in the mouse brain that is described as the human equivalent of the pregenual anterior cingulate cortex (pACC, Brodmann area 24), an area that shows abnormal activation in patients with depression is an interesting target for neuromodulation (Bicks et al., 2015). Overall, these data indicate that morphological plasticity in prefrontal areas is a biological mechanism of pivotal importance for alleviating symptoms of depression. We previously demonstrated that a short, 15 Hz, rTMS treatment modulates motor cortex spine density and dendritic morphology (Cambiaghi et al., 2021). Therefore, it is possible that HF-rTMS may alter mPFC synaptic architecture.

We assessed the effect of a 5-day HF-rTMS stimulation on emotional behavior, spine density, and dendritic complexity in the mPFC in mice. Our hypothesis was that a short HF-rTMS treatment should decrease immobility (antidepressant-like activity) when rodents are exposed to an inescapable situation coupled with synaptic rearrangements in layer II/III and layer V of the mPFC.

## Methods

### Animals

Forty 129/SvEv male mice (8 weeks old) were purchased from Taconic (Germantown, NY). Mice were provided with food and water *ad libitum* and kept on a 12:12 light/dark cycle (lights on at 7:00 am). The mice became accustomed to their habitat for 1 week before the beginning of the experiments. The protocol was approved by the Animal Care and Use Committee of the University of Verona (CIRSAL) and authorized by the Italian Ministry of Health (n. 718/2019-PR).

### Stimulation

**Figure 1:**
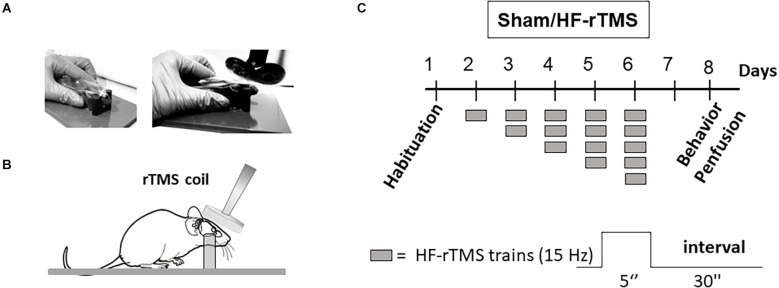
High-frequency-rTMS (HF-rTMS) stimulation and experimental design. **(A)** Representative picture of the awake mice restrained by a soft plastic funnel and a **(B)** graphic representation of the stimulation setup. **(C)** Schematic of the experimental timeline and HF-rTMS stimulation protocol. Awake mice restrained with a soft plastic funnel were stimulated with a rodent figure-eight coil. Golgi-stained slices were used to analyze dendritic spine density and complexity of layer II/III and V in the mPFC, prelimbic area.

Animals were divided into two groups, the sham condition (*n* = 20) and the real stimulation with HF-rTMS (*n* = 20). In experiments, rTMS was applied to awake mice by using a MagStim Rapid stimulator connected to a 25 mm figure-eight rodent coil (MagStim, Whitland, Dyfed, UK). The mice were restrained in a soft plastic funnel with the head positioned between two plastic bars to allow consistent positioning of the TMS coil. For the real stimulation, the coil was held in place using a dedicated plastic support with the center of the coil right above the frontal areas perpendicular to the mouse’s body axis (Cambiaghi et al., 2021; Figures 1A,B). For the sham condition, the coil was oriented away from the mouse’s head, and it was positioned about 30 cm from the mouse. We first determined the individual motor threshold, starting at 50% of the maximum stimulator output. The minimal stimulation intensity which induced a bilateral muscle twitch in the lower extremities (visually ascertained by the experimenter) was considered the motor threshold. The average motor threshold value was 50 ± 3.7% of the stimulator output. We applied HF-rTMS stimulation trains (5 s duration, 75 stimuli each train) separated by 30 s intervals. The stimulation took place for five consecutive days with a progressive increase in stimulation trains (one train on day 1 up to five trains on day 5). The day before the stimulation or the sham condition (day 1), mice were restrained in a plastic funnel for 2 min to allow them to familiarize with the experimental setting and procedure. The behavioral test and animal perfusion were performed 24 h after the last stimulation (Figure 1C).

### Behavioral tests

We assessed both anxiety-like behaviors (Open field Test-OFT, and Elevated-plus maze-EPM) and depression-like behaviors (Forced-swim test-FST, and Tail-Suspension Test-TST) following a previously described methodology (Crupi et al., 2010). A total of 30 animals were tested (two groups: Sham, *N* = 15; HF-rTMS, *N* = 15). Briefly, for the OFT we used a 50 × 50 arena. The floor was divided into 16 squares; the central four squares were defined as ‘the center “and the 12 squares alongside the walls as” the periphery’). Mice were placed in the center and videotaped for 5 min. Locomotor activity (number of lines crossing) and the time spent in the center were visually scored off-line. For the EPM, mice were placed for 5 min in a plus sign-shaped structure consisting of two opposing open arms (30 × 5 cm) and two opposing closed arms (30 × 5 × 15 cm) connected by a central platform (5 × 5 cm). The behavior was videotaped, and we scored the percentage of open-arms entries and time spent in the open arms. The FST was performed in a 14 cm diameter vertical glass cylinder, filled with water at 23 ± 2°C. Each mouse remained in the cylinder for 6 min, and the duration of immobility (floating) in the last 4 min was scored. For the TST, mice were suspended from the tail for 6 min with adhesive tape attached to a horizontal bar. The total duration of immobility was scored. All the behavioral tests were performed on the same day with a 2-h break between tests. The tests were performed in a randomized order.

### Golgi staining, spine density, and dendritic morphometric analysis

To investigate the effect of the stimulation protocol on mPFC morphological plasticity a group of 10 mice (Sham, *N* = 5 and HF-rTMS, *N* = 5) were sacrificed 24 h after the final rTMS session. Under deep anesthesia (Ketamine/Xylazine), mice were transcardially perfused with 4% paraformaldehyde in PBS and the brains were then immediately removed, postfixed for 24 h, and stained with FD Rapid GolgiStain Kit (FD NeuroTechnologies, Ellicott City, MD, USA). The brains were stored in the dark in solutions A + B for 2 weeks and for 48 h in solution C at 4°C. Then, the samples were frozen in dry ice and stored at −70°C until sectioning. Brain slices were cut in the cryostat to produce 100 μm sections which were mounted on slides which were then stained in a solution containing silver nitrate and covered with cover slips. Golgi-stained pyramidal cells of layer II/III and layer V (*n* = 10 neurons × brain × group) in the mPFC area from at least three independent coronal sections (between +2.10 and 1.78 mm relative to Bregma (Paxinos and Watson, 2013) were analyzed. Prelimbic layers were identified by their morphology and distance from the pial surface. Secondary basal and tertiary apical dendrites were examined by counting the number of spines. Under oil immersion through a 100× magnification on a microscope (Olympus BX63) spines were manually counted by an experimenter without the knowledge of experimental conditions (blind analysis). The spine density of each neuron was calculated by dividing the total number of spines counted on a neuron by the total length of the analyzed dendrite and expressed as the number of spines/μm dendrite. The mean spine density for each mouse was then calculated (Cambiaghi et al., 2021). The Sholl analysis was performed to investigate dendritic complexity. For each neuron, the three-dimensional basal and apical dendritic tree, including all branches, was reconstructed in a two-dimensional plane using a 40× magnification (Neurolucida; MicroBrighField, Williston, VT). The dendritic tracing was analyzed using the NeuroExplorer software (MicroBrightField) to quantify dendritic complexity by measuring dendritic length and number of intersections (branch points) at a different radius from the soma center (10 μm steps). Samples were number-coded and blind analysis was performed (Cambiaghi et al., 2020, 2021).

### Statistical analysis

The data were analyzed with the GraphPad Prism 8 software (GraphPad Software, CA, USA). Group differences were analyzed with an unpaired, two-tailed t-test. Dendritic complexity was assessed with a two-way ANOVA (main effects: treatment and radius) followed by Bonferroni’s correction. *P* < 0.05 was considered statistically significant.

## Results

**Figure 2:**
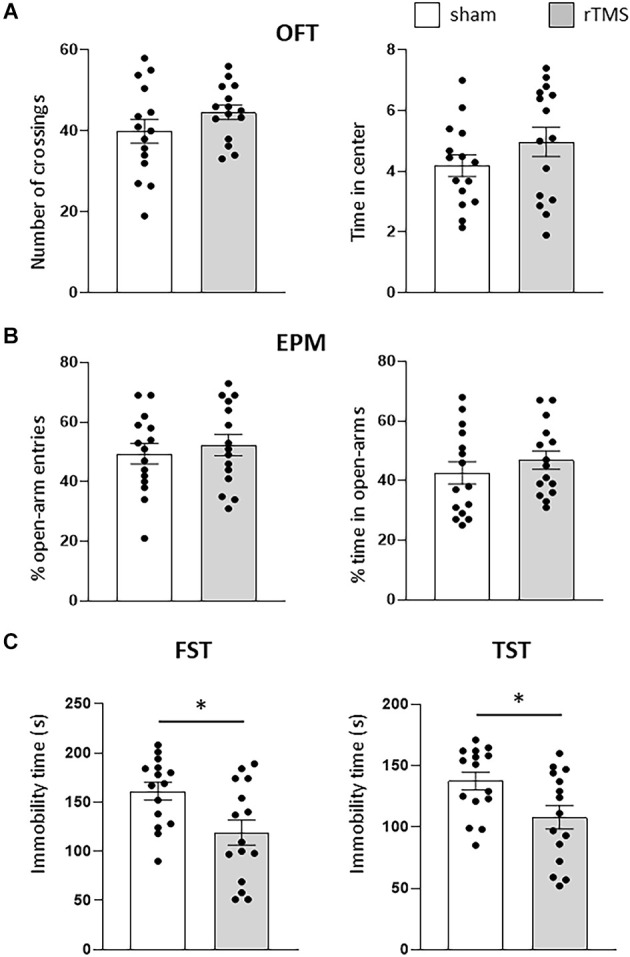
HF-rTMS modifies depressive-like behavior. In the anxiety-like tests, stimulated and sham animals did not show any difference in the open field test (OFT) **(A)**, when measuring both the locomotor activity (number of crossing) and time spent in the center of the arena. Similar results were obtained by analyzing the entries and time spent in the open arms of the elevated plus maze (EPM) test **(B)**. In the depression-like tests, a significant decrease in immobility time was found in both the forced swim test (FST) and in the tail suspension test (TST) **(C)**. Means ± SE. *p <0.05.

We initially used anxiety-like and depression-like behavioral tests (OFT, EPM, FST, and TST) to evaluate the effects of the HF-rTMS treatment on animals. Stressed and anxious mice tend to spend less time in both the center of the OFT arena and in the open arms of the EPM. HF-rTMS stimulated mice spent the same amount of time in the center of the arena as the sham mice (4.1 ± 0.4 vs. 4.9 ± 0.5 s; *p* = 0.27), with a similar locomotor activity, as assessed by analyzing the number of crossings (44.6 ± 2.4 vs. 39.8 ± 4 ; *p* = 0.33; Figure 2A). In the EPM, stimulated mice showed similar anxiety levels as the sham group, as evaluated by both the percentage of open-arm entries (52.4 ± 4.2% vs. 49.3 ± 4.3%, *p* = 0.61) and time in the open arms (46.8 ± 3.9 vs. 42.6 ± 4.3%, ; *p* = 0.48; Figure 2B). Therefore, these results indicate that the stimulation protocol did not lead to increased anxiety. We then examined the effect of the treatment on depressive-like behavior. In the FST, the immobility time was significantly lower in the stimulated group (118.9 ± 13.4 vs. 162 ± 11.4 s; *p* = 0.025). Similar results were obtained in the TST (107.8 ± 10.2 vs. 137.1 ± 7 s ; *p* > 0.05; Figure 2C). Therefore, the HF-rTMS treatment induced a significant antidepressant-like effect.

**Figure 3:**
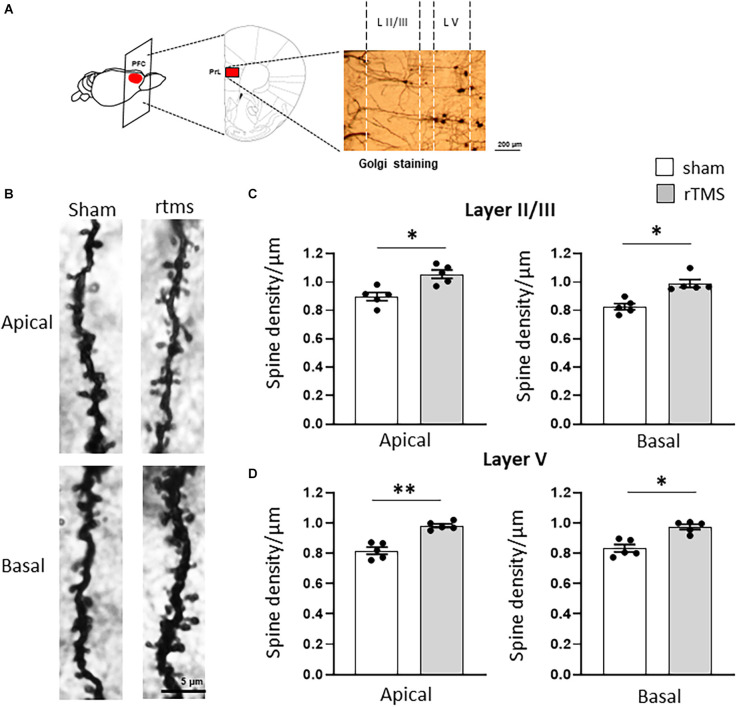
HF-rTMS modulates apical and basal dendritic spines in layer II/III prefrontal neurons within the prelimbic area. **(A)** Schematic of the stimulated brain area and representative Golgi staining of the analyzed region of interest (scale bar = 200 μm). **(B)** Representative images of apical and basal dendrites for the sham and rTMS conditions (L II/III). Stimulation resulted in a significantly increased spine density in both apical and basal dendrites of layer II/III **(C)** and layer V **(D)** (scale bar = 5 μm). Means ± SE. *p < 0.05, **p < 0.01.

**Figure 4:**
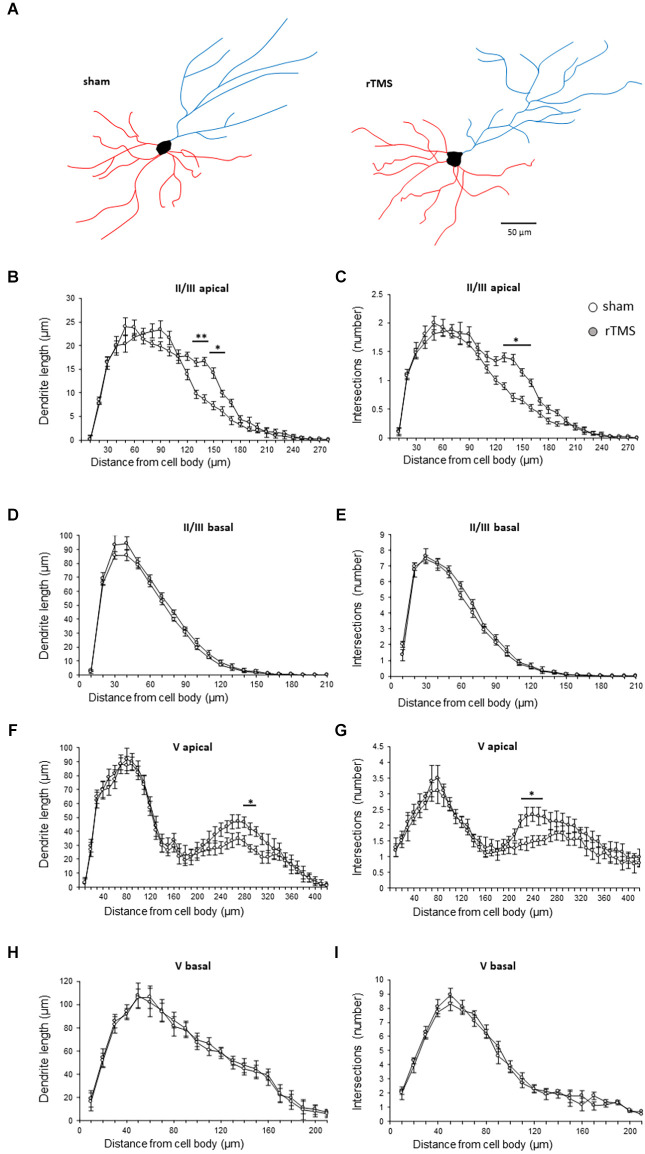
HF-rTMS induced dendritic plasticity medial prefrontal cortex layer II/III and V pyramidal neurons apical dendrites. **(A)** Schematic representation of reconstructed layer II/III neurons for the sham and HF-rTMS groups (scale bar = 50 mm). Quantification of dendritic length and the number of intersections in both layer II/III pyramidal neurons apical **(B,C)** and basal **(D,E)** dendrites and in layer V pyramidal neurons apical **(F,G)** and basal **(H,I)** dendrites. Means ± SE. *p < 0.05, **p < 0.01.

We then investigated whether the observed behavioral changes were associated with a structural change in the dendritic morphology of mPFC pyramidal neurons. We used Golgi-Cox staining to identify individual mPFC layer II/III and layer V pyramidal neurons (Figures 3A,B) and analyzed both the dendritic spine density and the dendritic tree complexity by using the Sholl analysis. The dendritic spine counts of layer II/III revealed a significant effect of the HF-rTMS in both apical and basal arborization (*p* = 0.020 and *p* = 0.011 respectively; Figure 3C). Similar changes occurred in layer V at both apical and basal dendrites (*p* = 0.004 and *p* = 0.025 respectively; Figure 3D). The Sholl analysis (Figure 4A) revealed that the HF-rTMS had a significant effect on the complexity of apical dendrites in both layers. In layer II/III, a two-way ANOVA analysis (factors: radius and treatment) indicated a difference of both factors and their interaction for the length (main effect treatment: *F*_(1,8)_ = 16.01, *p* = 0.0039; radius: *F*_(4.1,33.2)_ = 199.6, *p* < 0.0001, treatment × radius interaction: *F*_(28,224)_ = 4.714, *p* < 0.0001) and the number of intersections (main effect treatment: *F*_(1,8)_ = 10.76, *p* = 0.011; radius: *F*_(4.5,36.1)_ = 196.3, *p* < 0.0001, treatment × radius interaction: *F*_(28,224)_ = 4.114, *p* < 0.0001). Bonferroni’s correction showed that the increase in length in the stimulated group is in the range of 130–160 μm (*p* = 0.0048; *p* = 0.0035; *p* = 0.0352; *p* = 0.0439; Figure 4B). Furthermore, treated animals showed an increase in the number of nodes in the same range (*p* = 0.0343; *p* = 0.0176; *p* = 0.0304; *p* = 0.0223; Figure 4C). By contrast, basal dendrite branching showed no difference between the groups in both length (main effect treatment: *F*_(1,8)_ = 0.38, *p* = 0.55; radius: *F*_(2.1,16.8)_ = 534.3, *p* < 0.0001, treatment × radius interaction: *F*_(26,208)_ = 0.804, *p* = 0.69; Figure 4D) and number of intersections (main effect treatment: *F*_(1,8)_ = 0.04, *p* = 0.83; radius: *F*_(3.3,26.6)_ = 464.0, *p* < 0.0001, treatment × radius interaction: *F*_(26,208)_ = 0.652, *p* = 0.902; Figure 4E). When we analyzed the dendritic complexity of layer V, we found an effect of the stimulation on apical dendrite length (main effect treatment: *F*_(1,8)_ = 0.48, *p* = 0.69; radius: *F*_(3.8,31.7)_ = 311.8, *p* < 0.0001, treatment × radius interaction: *F*_(42,336)_ = 3.711, *p* < 0.0001) and intersections (main effect treatment: *F*_(1,8)_ = 0.59, *p* = 0.53; radius: *F*_(2.7,26.6)_ = 402.1, *p* < 0.0001, treatment × radius interaction: *F*_(42,336)_ = 4.027, *p* < 0.0001). The Bonferroni’s analysis revealed that stimulated animals had an increased length in the radius interval between 280 and 300 μm (*p* = 0.041: *p* = 0.025; *p* = 0.016; Figure 4F) while the increase in the number of intersections took place between 230 and 260 μm (*p* = 0.044: *p* = 0.018; *p* = 0.041; *p* = 0.022; Figure 4G). Similar to layer II/III, the two-way ANOVA analysis did not show a significant interaction for both the basal arborization length (main effect treatment: *F*_(1,8)_ = 0.21, *p* = 0.87; radius: *F*_(5.1,19.4)_ = 371.8, *p* < 0.0001, treatment × radius interaction: *F*_(26,208)_ = 0.715, *p* = 0.78) and the number of nodes (main effect treatment: *F*_(1,8)_ = 0.34, *p* = 0.67; radius: *F*_(4.6,14.2)_ = 389.5, *p* < 0.0001, treatment × radius interaction: *F*_(26,208)_ = 0.848, *p* = 0.62; Figures 4H,I).

## Discussion

In this study, we showed that the HF-rTMS treatment we used improved depression-like behaviors, increased dendritic spine density, and dendritic arborization of neurons in both superficial and deep layers of the mouse mPFC.

Growing evidence indicates that mPFC plays a pivotal role in depressive-like behavior. Our results are in keeping with previous studies indicating that different HF-rTMS protocols induce antidepressant-like effects in rodents (Yang et al., 2007; Feng et al., 2012; Wang et al., 2014; Heath et al., 2018; Yan et al., 2022). Moreover, the protocols used in the aforementioned studies required longer stimulation, immobilization, and handling of animals which induce stress and a consequent detrimental effect on morphological and functional plasticity (Herman et al., 2003). Our results have shown that a short HF-rTMS protocol does not increase stress in mice and is still capable of inducing antidepressant-like effects. Thus, our protocol may be suited to assess the effect of plasticity-based antidepressant treatments on morphological biomarkers in rodents. Although the different brain circuits engaged during exposure to inescapable stressful situations like TST and FST are still to be fully elucidated, the rodent mPFC receives inputs from several brain regions and projects to key areas regulating mood, emotion, and stress response (Laubach et al., 2018). For instance, the networks connecting the mPFC with the dorsal raphe nucleus (DRN) serotonergic neurons, the ventral hippocampus, and the nucleus accumbens (Nac) are thought to be involved in antidepressant-like effects in rodents (Warden et al., 2012; Wang et al., 2014; Urban et al., 2016). Furthermore, previous works have detailed the role of the balance between mPFC glutamatergic/excitatory and GABAergic/inhibitory circuits in mood regulation induced by both HF-rTMS and the rapid acting antidepressants such as ketamine (Fee et al., 2017; Mineo et al., 2018; Yin et al., 2021). The use of optogenetic and chemogenetic techniques may further elucidate at the cellular level the mechanisms of action underlying the behavioral gain induced by HF-rTMS treatment. For instance, photostimulation of mPFC glutamatergic neurons receiving input from the DRN reduces immobility time in the TST (Warden et al., 2012). In the same way, stimulation of the afferents from the ventral hippocampal to the mPFC reduces immobility time in the FST (Carreno et al., 2016). Future studies are needed to investigate the interplay between genetic and environmental factors and to test whether a similar behavioral gain may occur in different mouse strains (Abramov et al., 2008). Since in our study we used a figure-of-eight rodent coil which stimulates multiple mouse brain areas, it is likely that multiple networks contribute to the emotional behavioral modulation induced by HF-rTMS.

Moreover, we showed that behavioral changes were paralleled by morphological plastic changes in mPFC pyramidal neurons, with a significant increase in spine density in both layers II/III and V, apical and basal dendrites. Regarding dendritic complexity, the stimulated mice showed increased dendritic length and number of intersections only in the apical dendrites. Previous studies indicate that spine density is proportional to the excitatory synaptic input to a given neuron (Hering and Sheng, 2001). Patients with major depression show low levels of glutamate in prefrontal cortex regions (Arnone et al., 2015) and antidepressants increase glutamate concentration in frontal areas (Stone et al., 2012; Wojtas et al., 2022). Hence, HF-rTMS might exert positive effects on depression-like behavior through the regulation of glutamatergic neurotransmission in mPFC and a consequent increase in spine density and dendritic complexity. Additionally, mounting evidence suggests a key role of brain-derived neurotrophic factor (BDNF) in mediating the antidepressant-like effects of HF-rTMS (Vlachos et al., 2012). BDNF is a regulator of synaptic plasticity in the brain and may be a key molecule involved in the mechanism underlying the relationship between HF-rTMS and morphological plasticity in mPFC. In this context, it is worth noting that a low level of BDNF is strongly associated with depression and antidepressant drugs can normalize BDNF levels (Castrén and Monteggia, 2021). Furthermore, mice with forebrain BDNF alterations are known to display impaired spine-synapse number and dendrite complexity (Duman et al., 2021). It is recognized that BDNF exerts a key role in the induction of long-term potentiation (LTP) in the adult mouse brain. Since cortical LTP is associated with increases in spine density and dendritic arborization in the cortex (Muller et al., 2000; Monfils et al., 2004), it is likely that BDNF may play a pivotal role in mediating the antidepressant-like effect of HF-rTMS.

Our treatment resulted in a significant modulation only on the apical dendrites, mainly in their medial-to-distal portion. This finding is in agreement with other studies indicating that stress induces a significant reorganization of apical dendrites in layer II/III pyramidal neurons of the mPFC, but not in the basal dendrites (Wellman, 2001; Radley et al., 2004), suggesting that HF-rTMS may counteract the negative effect of stress on mPFC morphology.

A limitation of the study is the low focality of the stimulation delivered through the rodent coil, which may have induced changes in different brain regions. It is possible that neuromodulation of sensory and motor brain areas might play a role in determining behavioral gain since in both the FST and TST mice will first make efforts to escape but eventually will exhibit immobility which reflects a measure of behavioral despair. Furthermore, the determination of the motor threshold in awake mice using rodent coils is a rather gross measure. Thus, our results should be confirmed in future studies using a more focal stimulation, a better determination of the individual motor threshold (using electromyographic recoding in anesthetized mice), and protocols should ascertain the effect in animal models of depression. In addition, the rodent mPFC has different interconnected subregions, receiving several inputs and outputs and, in future works, it will be important to explore in detail the effects of focal stimulation of each subregion using optogenetic techniques. We have emphasized a possible pivotal role for growth factors; we should also state that modulation of neurotransmitters, receptors, and inhibitory circuits could play a significant contribution to the behavioral effects we are reporting in our study.

Lastly, this was a pilot study and we have used only male mice because of a previously published study that addressed cortical morphological plasticity in a different brain area using the same protocol and the same mouse strain (Cambiaghi et al., 2021). This approach allowed us to discuss region-specific effects. We plan to confirm and expand our findings using both female and male mice.

In conclusion, the present study expands the previous literature and furthers our understanding of the biological mechanisms underlying the therapeutic effects of HF-rTMS to show a pivotal role for structural plastic changes within the mPFC. Future studies are required to better understand mPFC neuromodulation in clinical and rodent models of depression, aging, and dementia.

## Data Availability Statement

The raw data supporting the conclusions of this article will be made available by the authors, without undue reservation.

## Ethics Statement

The animal study was reviewed and approved by University of Verona (CIRSAL) and authorized by the Italian Ministry of Health (n. 718/2019-PR).

## Author Contributions

FB: conceptualization, project administration, and resources. MC, CI, and FB: data acquisition. MC, CI, FG, AE, WM, and MB: software and data analysis. MC, CI, FG, AE, WM, MB, ZH, RS, FT, and FB: writing the original drafts. MC, CI, ZH, RS, FT, and FB: writing—review and editing. All authors contributed to the article and approved the submitted version.

## References

[B1] Abramov U. Puussaar T. Raud S. Kurrikoff K. Vasar E. (2008). Behavioural differences between C57BL/6 and 129S6/SvEv strains are reinforced by environmental enrichment. Neurosci. Lett. 443, 223–227. 10.1016/j.neulet.2008.07.075 18687379

[B302] Allen J. J. Urry H. L. Hitt S. K. Coan J. A. (2004). The stability of resting frontal electroencephalographic asymmetry in depression. Psychophysiology 41, 269–280. 10.1111/j.1469-8986.2003.00149.x 15032992

[B2] Arnone D. Mumuni A. N. Jauhar S. Condon B. Cavanagh J. (2015). Indirect evidence of selective glial involvement in glutamate-based mechanisms of mood regulation in depression: meta-analysis of absolute prefrontal neuro-metabolic concentrations. Eur. Neuropsychopharmacol. 25, 1109–1117. 10.1016/j.euroneuro.2015.04.016 26028038

[B3] Barker A. T. Jalinous R. Freeston I. L. (1985). Non-invasive magnetic stimulation of human motor cortex. Lancet 1, 1106–1107. 10.1016/s0140-6736(85)92413-4 2860322

[B4] Bicks L. K. Koike H. Akbarian S. Morishita H. (2015). Prefrontal cortex and social cognition in mouse and man. Front. Psychol. 6:1805. 10.3389/fpsyg.2015.01805 26635701 PMC4659895

[B5] Cambiaghi M. Cherchi L. Masin L. Infortuna C. Briski N. Caviasco C.. (2021). High-frequency repetitive transcranial magnetic stimulation enhances layer II/III morphological dendritic plasticity in mouse primary motor cortex. Behav. Brain Res. 410:113352. 10.1016/j.bbr.2021.113352 33979657

[B6] Cambiaghi M. Crupi R. Bautista E. L. Elsamadisi A. Malik W. Pozdniakova H.. (2020). The effects of 1-Hz rTMS on emotional behavior and dendritic complexity of mature and newly generated dentate gyrus neurons in male mice. Int. J. Environ. Res. Public Health 17:4074. 10.3390/ijerph17114074 32521613 PMC7312937

[B7] Carreno F. R. Donegan J. J. Boley A. M. Shah A. Deguzman M. Frazer A.. (2016). Activation of a ventral hippocampus-medial prefrontal cortex pathway is both necessary and sufficient for an antidepressant response to ketamine. Mol. Psychiatry 21, 1298–1308. 10.1038/mp.2015.176 26619811

[B8] Castrén E. Monteggia L. M. (2021). Brain-derived neurotrophic factor signaling in depression and antidepressant action. Biol. Psychiatry 90, 128–136. 10.1016/j.biopsych.2021.05.008 34053675

[B9] Covington H. E. 3rd Lobo M. K. Maze I. Vialou V. Hyman J. M. Zaman S.. (2010). Antidepressant effect of optogenetic stimulation of the medial prefrontal cortex. J. Neurosci. 30, 16082–16090. 10.1523/JNEUROSCI.1731-10.2010 21123555 PMC3004756

[B10] Crupi R. Cambiaghi M. Spatz L. Hen R. Thorn M. Friedman E.. (2010). Reduced adult neurogenesis and altered emotional behaviors in autoimmune-prone B-cell activating factor transgenic mice. Biol. Psychiatry 67, 558–566. 10.1016/j.biopsych.2009.12.008 20185032

[B11] Csabai D. Wiborg O. Czéh B. (2018). Reduced synapse and axon numbers in the prefrontal cortex of rats subjected to a chronic stress model for depression. Front. Cell. Neurosci. 12:24. 10.3389/fncel.2018.00024 29440995 PMC5797661

[B303] Davidson R. J. (1992). Anterior cerebral asymmetry and the nature of emotion. Brain Cogn. 20, 125–151. 10.1016/0278-2626(92)90065-t 1389117

[B12] Dodd S. Mitchell P. B. Bauer M. Yatham L. Young A. H. Kennedy S. H.. (2018). Monitoring for antidepressant-associated adverse events in the treatment of patients with major depressive disorder: an international consensus statement. World J. Biol. Psychiatry 19, 330–348. 10.1080/15622975.2017.1379609 28984491

[B13] Duman R. S. Aghajanian G. K. Sanacora G. Krystal J. H. (2016). Synaptic plasticity and depression: new insights from stress and rapid-acting antidepressants. Nat. Med. 22, 238–249. 10.1038/nm.4050 26937618 PMC5405628

[B14] Duman R. S. Deyama S. Fogaça M. V. (2021). Role of BDNF in the pathophysiology and treatment of depression: activity-dependent effects distinguish rapid-acting antidepressants. Eur. J. Neurosci. 53, 126–139. 10.1111/ejn.14630 31811669 PMC7274898

[B15] Fee C. Banasr M. Sibille E. (2017). Somatostatin-positive gamma-aminobutyric acid interneuron deficits in depression: cortical microcircuit and therapeutic perspectives. Biol. Psychiatry 82, 549–559. 10.1016/j.biopsych.2017.05.024 28697889 PMC5610074

[B16] Feng S. F. Shi T. Y. Fan Y. Wang W. N. Chen Y. C. Tan Q. R. (2012). Long-lasting effects of chronic rTMS to treat chronic rodent model of depression. Behav. Brain Res. 232, 245–251. 10.1016/j.bbr.2012.04.019 22537774

[B17] George M. S. Caulfield K. A. Wiley M. (2022). Shaping plasticity with non-invasive brain stimulation in the treatment of psychiatric disorders: Present and future. Handb. Clin. Neurol. 184, 497–507. 10.1016/B978-0-12-819410-2.00028-X 35034757 PMC9985830

[B307] George M. S. Lisanby S. H. Avery D. McDonald W. M. Durkalski V. (2010). Daily left prefrontal transcranial magnetic stimulation therapy for major depressive disorder: a sham-controlled randomized trial. Arch. Gen. Psychiatry 67, 507–516. 10.1001/archgenpsychiatry.2010.46 20439832

[B18] Heath A. Lindberg D. R. Makowiecki K. Gray A. Asp A. J. Rodger J.. (2018). Medium- and high-intensity rTMS reduces psychomotor agitation with distinct neurobiologic mechanisms. Transl. Psychiatry 8:126. 10.1038/s41398-018-0129-3 29976924 PMC6033856

[B19] Hering H. Sheng M. (2001). Dendritic spines: structure, dynamics and regulation. Nat. Rev. Neurosci. 2, 880–888. 10.1038/35104061 11733795

[B20] Herman J. P. Figueiredo H. Mueller N. K. Ulrich-Lai Y. Ostrander M. M. Choi D. C.. (2003). Central mechanisms of stress integration: hierarchical circuitry controlling hypothalamo-pituitary-adrenocortical responsiveness. Front. Neuroendocrinol. 24, 151–180. 10.1016/j.yfrne.2003.07.001 14596810

[B21] Holmes S. E. Abdallah C. Esterlis I. (2022). Imaging synaptic density in depression. Neuropsychopharmacology. 10.1038/s41386-022-01368-4. [Online ahead of print]. 35768568 PMC9700860

[B22] Laubach M. Amarante L. M. Swanson K. White S. R. (2018). What, if anything, is rodent prefrontal cortex? eNeuro 5:ENEURO.0315-18.2018. 10.1523/ENEURO.0315-18.2018 30406193 PMC6220587

[B23] Liu R. J. Aghajanian G. K. (2008). Stress blunts serotonin- and hypocretin-evoked EPSCs in prefrontal cortex: role of corticosterone-mediated apical dendritic atrophy. Proc. Natl. Acad. Sci. U S A 105, 359–364. 10.1073/pnas.0706679105 18172209 PMC2224217

[B24] Marcus D. J. Bedse G. Gaulden A. D. Ryan J. D. Kondev V. Winters N. D.. (2020). Endocannabinoid signaling collapse mediates stress-induced amygdalo-cortical strengthening. Neuron 105, 1062–1076.e6. 10.1016/j.neuron.2019.12.024 31948734 PMC7992313

[B25] Mineo L. Concerto C. Patel D. Mayorga T. Chusid E. Infortuna C.. (2018). Modulation of sensorimotor circuits during retrieval of negative Autobiographical Memories: exploring the impact of personality dimensions. Neuropsychologia 110, 190–196. 10.1016/j.neuropsychologia.2017.04.016 28404231

[B26] Monfils M. H. Vandenberg P. M. Kleim J. A. Teskey G. C. (2004). Long-term potentiation induces expanded movement representations and dendritic hypertrophy in layer V of rat sensorimotor neocortex. Cereb. Cortex 14, 586–593. 10.1093/cercor/bhh020 15054074

[B27] Muller D. Toni N. Buchs P. A. (2000). Spine changes associated with long-term potentiation. Hippocampus 10, 596–604. 10.1002/1098-1063(2000)10:5<596::AID-HIPO10>3.0.CO;2-Y 11075830

[B28] Paxinos G. Watson C. (2013). The Rat Brain in Stereotaxic Coordinates. San Diego, CA: Academic Press.

[B29] Radley J. J. Sisti H. M. Hao J. Rocher A. B. Mccall T. Hof P. R.. (2004). Chronic behavioral stress induces apical dendritic reorganization in pyramidal neurons of the medial prefrontal cortex. Neuroscience 125, 1–6. 10.1016/j.neuroscience.2004.01.006 15051139

[B30] Riga D. Matos M. R. Glas A. Smit A. B. Spijker S. Van Den Oever M. C. (2014). Optogenetic dissection of medial prefrontal cortex circuitry. Front. Syst. Neurosci. 8:230. 10.3389/fnsys.2014.00230 25538574 PMC4260491

[B31] Somani A. Kar S. K. (2019). Efficacy of repetitive transcranial magnetic stimulation in treatment-resistant depression: the evidence thus far. Gen. Psychiatry 32:e100074. 10.1136/gpsych-2019-100074 31552384 PMC6738665

[B32] Stone J. M. Dietrich C. Edden R. Mehta M. A. De Simoni S. Reed L. J.. (2012). Ketamine effects on brain GABA and glutamate levels with 1H-MRS: relationship to ketamine-induced psychopathology. Mol. Psychiatry 17, 664–665. 10.1038/mp.2011.171 22212598 PMC3883303

[B33] Urban D. J. Zhu H. Marcinkiewcz C. A. Michaelides M. Oshibuchi H. Rhea D.. (2016). Elucidation of the behavioral program and neuronal network encoded by dorsal raphe serotonergic neurons. Neuropsychopharmacology 41, 1404–1415. 10.1038/npp.2015.293 26383016 PMC4793125

[B34] Vialou V. Bagot R. C. Cahill M. E. Ferguson D. Robison A. J. Dietz D. M.. (2014). Prefrontal cortical circuit for depression- and anxiety-related behaviors mediated by cholecystokinin: role of ΔFosB. J. Neurosci. 34, 3878–3887. 10.1523/JNEUROSCI.1787-13.2014 24623766 PMC3951691

[B35] Vlachos A. Müller-Dahlhaus F. Rosskopp J. Lenz M. Ziemann U. Deller T. (2012). Repetitive magnetic stimulation induces functional and structural plasticity of excitatory postsynapses in mouse organotypic hippocampal slice cultures. J. Neurosci. 32, 17514–17523. 10.1523/JNEUROSCI.0409-12.2012 23197741 PMC6621866

[B36] Wang H. N. Wang L. Zhang R. G. Chen Y. C. Liu L. Gao F.. (2014). Anti-depressive mechanism of repetitive transcranial magnetic stimulation in rat: the role of the endocannabinoid system. J. Psychiatr. Res. 51, 79–87. 10.1016/j.jpsychires.2014.01.004 24479995

[B37] Warden M. R. Selimbeyoglu A. Mirzabekov J. J. Lo M. Thompson K. R. Kim S. Y.. (2012). A prefrontal cortex-brainstem neuronal projection that controls response to behavioural challenge. Nature 492, 428–432. 10.1038/nature11617 23160494 PMC5929119

[B38] Wellman C. L. (2001). Dendritic reorganization in pyramidal neurons in medial prefrontal cortex after chronic corticosterone administration. J. Neurobiol. 49, 245–253. 10.1002/neu.1079 11745662

[B39] Williams L. M. (2016). Precision psychiatry: a neural circuit taxonomy for depression and anxiety. Lancet Psychiatry 3, 472–480. 10.1016/S2215-0366(15)00579-9 27150382 PMC4922884

[B40] Wojtas A. Bysiek A. Wawrzczak-Bargiela A. Szych Z. Majcher-Maślanka I. Herian M.. (2022). Effect of psilocybin and ketamine on brain neurotransmitters, glutamate receptors, DNA and rat behavior. Int. J. Mol. Sci. 23:6713. 10.3390/ijms23126713 35743159 PMC9224489

[B41] Yan J. Zhang F. Niu L. Wang X. Lu X. Ma C.. (2022). High-frequency repetitive transcranial magnetic stimulation mitigates depression-like behaviors in CUMS-induced rats via FGF2/FGFR1/p-ERK signaling pathway. Brain Res. Bull. 183, 94–103. 10.1016/j.brainresbull.2022.02.020 35247488

[B42] Yang Y. Li W. Zhu B. Liu Y. Yang B. Wang H.. (2007). Sex differences in antidepressant-like effect of chronic repetitive transcranial magnetic stimulation in rats. Prog. Neuropsychopharmacol. Biol. Psychiatry 31, 735–740. 10.1016/j.pnpbp.2007.01.007 17291659

[B43] Yin Y. Y. Wang Y. H. Liu W. G. Yao J. Q. Yuan J. Li Z. H.. (2021). The role of the excitation: inhibition functional balance in the mPFC in the onset of antidepressants. Neuropharmacology 191:108573. 10.1016/j.neuropharm.2021.108573 33945826

